# A scoping review of psychological distress instruments in women with early‐stage breast cancer during chemotherapy

**DOI:** 10.1002/cnr2.1833

**Published:** 2023-05-12

**Authors:** Amal Khulaif Alanazi, Debra Lynch‐Kelly, Michael Weaver, Debra E. Lyon

**Affiliations:** ^1^ College of Nursing King Saud bin Abdulaziz University for Health Sciences Riyadh Saudi Arabia; ^2^ Department of Biobehavioral Nursing Science College of Nursing, University of Florida Gainesville Florida USA

**Keywords:** breast cancer, chemotherapy, oncology, psychological distress, scoping review, stress

## Abstract

**Background:**

Psychological distress is associated with worsening symptoms during the active treatment period and lower quality of life in women with early‐stage breast cancer. Many studies have indicated risk for heightened psychological distress across the breast cancer trajectory.

**Purpose:**

The aim of this review is to examine the literature for instruments used to measure psychological distress among women with breast cancer during chemotherapy.

**Methods:**

This study used the Arksey and O’Malley framework of scoping reviews. Two databases, PubMed & CINAHL, were searched for peer‐reviewed original articles that were published within the last ten years, included participants with a diagnosis of breast cancer stages I to III, and receiving chemotherapy, English text articles, and studies that report psychological distress measures.

**Findings:**

The initial screening yielded 529 relevant studies. After applying the exclusion criteria, a total of 17 studies concerning the assessment of psychological distress during chemotherapy were retained for the analysis of variables and measures of psychological distress. The instruments used to measure psychological distress varied, with a total of 21 measures. The most frequently utilized measure was the Hospital Anxiety and Depression Scale (*n* = 5), followed by the Impact of Event Scale (*n* = 2), the Distress Thermometer (*n* = 2), and the Perceived Stress Scale (*n* = 2).

**Conclusion:**

This review identified the gaps related to inconsistencies in the operationalization and instruments used to measure psychological distress among breast cancer survivors during chemotherapy. Standardization of measures assessing psychological distress, along with conceptual clarity, is essential for measuring distress in research and clinical practice.

## 1 BACKGROUND

Breast cancer (BC) is the most common cancer type among women worldwide.^1^ Furthermore, advances in medical care, such as early detection and treatment, have increased the 5‐year survival rate to 90%.^2^ Despite these huge improvements, a large proportion of breast cancer survivors (BCSs) experience periods of stress accompanying treatments, including chemotherapy, surgery, radiation, and endocrine medications.^3^, ^4^

The context of this review is focused mainly on the chemotherapy period, which is an important part of breast cancer treatment; however, it carries a high symptom burden.^5^ Recent studies reported that chemotherapy can result in varied symptoms, such as anxiety, depression, fatigue, and cognitive impairment.^4^, ^6^ Anxiety and depression have been frequently examined together during active treatment, and their incidence was commonly as high as 93.6% and 25%, respectively.^7^, ^8^ Furthermore, in symptom cluster research, fatigue has consistently been prevalent alongside symptoms of distress among women treated with chemotherapy.^9^, ^10^ Also, cognitive impairment is another comorbidity that has been correlated with other symptoms of distress during chemotherapy.^6^, ^10^ These symptoms dispose BCSs to have higher risk of psychological distress, poor daily function, and reduced life quality, even after the completion of chemotherapy.

Psychological distress is a broad construct that has varying definitions in the literature.^11^, ^12^ Most of the researchers have utilized multiple terms to describe psychological distress that have commonly included anxiety, depression, and distress.^13^, ^14^, ^15^ The American Psychological Association (APA) defines distress as “the negative form of stress in which a person experiences a negative emotional state that is usually underrecognized”.^16^ Furthermore, the APA has defined stress as a natural reaction to stressful life events and can cause an adverse effect if it persists for too long.^16^ However, when reported in relation to cancer and cancer‐related symptoms, stress is almost always synonymous with distress. Also, stress can negatively impact health, leading to immune downregulation that results in chronic health conditions such as heart disease, stomach ulcers, and renal disorders.^16^, ^17^ Thus, it is essential to assess to manage BCSs' symptoms during active therapy effectively.

The National Comprehensive Cancer Network (NCCN) recommends using the term distress to avoid the stigma associated with other mental disorders.^18^ Distress is anticipated during the survivorship period of breast cancer and can be mild if patients' symptoms are not severe and do not persist long after the diagnosis.^18^ The current guidelines recommend using the Distress Thermometer (DT) and Problem List (PL) scales for early detection of distress among cancer patients during each clinical visit.^1^, ^18^ The NCCN emphasized the role of clinicians in diagnosing moderate to severe psychological distress that is in line with the criteria found in the Diagnostic and Statistical Manual of Mental Disorders Fourth Edition, Text Revision (DSM‐IV‐TR). However, there is still a gap in the literature regarding the proper identification of psychological distress among breast cancer survivors.^19^, ^20^

Prior research has reported that 20% to 50% of women had psychological distress during their diagnosis and treatment for breast cancer.^15^, ^21^ Moreover, the impact of psychological distress can extend beyond treatment into the period of survivorship.^11^, ^14^ Distress among cancer patients can be related to treatment options, including surgery, chemotherapy, and radiation; it can also result from the side effects of these therapies, such as anxiety, nausea, and fatigue. These symptoms influence breast cancer patients' survivorship and quality of life.^6^, ^9^, ^22^

Previous reviews on psychological distress have described the impact of distress on young women and the predictors associated with distress.^19^, ^23^ Another review investigated the impacts of stress and chemotherapy on cognitive performance among breast cancer patients.^24^ However, the literature shows only a single review released by the National Breast Care Center (NBCC) that emphasizes the importance of early identification of distress in women with breast cancer.^25^ The NBCC's report was limited to self‐reported measures of psychological distress.

The purpose of this review was to examine the literature for instruments used to measure psychological distress among women with breast cancer during chemotherapy. To our knowledge, the present review is the first one that focuses on the assessment of psychological distress during chemotherapy treatment. Understanding measurements of distress in the literature is an essential step in future management of breast cancer patients' distressing symptoms. With more reviews like the present one, clinicians and healthcare providers may better understand screening measures for psychological distress to intervene and improve quality of life for women living with breast cancer.

## 2 METHODS

This review used a scoping review process of mapping the available literature covering this research area.^26^ In this review, we used the five stages outlined in the framework by Arksey and O'Malley, which include identifying the research question, identifying the relevant studies, selecting studies, charting the data, and collating, summarizing, and reporting the result.^27^ We used this framework to explore the literature on the measures of psychological distress among BCSs. We reported our findings using the Preferred Reporting Items for Systematic Reviews and Meta‐Analyses extension for Scoping Review (PRISMA‐ScR).^28^

### 2.1 Identifying the research question

The following questions were addressed by the literature search:

1. What instruments are used to measure psychological distress among breast cancer survivors?
2. What are the concepts that were used to define psychological distress?

### 2.2 Identifying relevant studies

A scoping review was conducted to map the existing literature on the instruments used to assess psychological distress for breast cancer survivors undergoing chemotherapy. In consultation with a research librarian, studies were identified using a systematic review of the literature available on PubMed and CINAHL within the 10 years leading up to July 5, 2019. The initial search strategy consisted of the terms “psychological stress” OR “stress” OR “distress” AND “drug therapy”; OR “drug therapy” OR “chemotherapy” OR “active treatment” AND “Breast Neoplasms”; OR “breast cancer” OR “breast neoplasm” OR “breast tumor” OR “breast carcinoma.” A second search was conducted using all the identified keywords, and Medical Subject Heading (MeSH) was applied when searching the databases. Also, due to the subjectivity of the term distress, and based on the description of distress, we decided to include the studies that reported psychological stress tools. Fully reproducible search strategies for each database and search are available in Supplemental File 1.

### 2.3 Study selection

**FIGURE 1:**
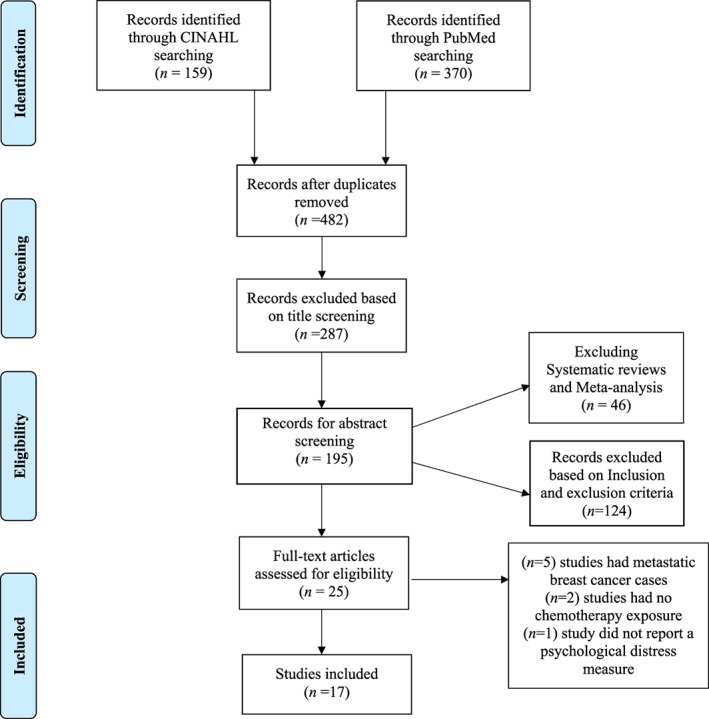
PRISMA flowchart of the studies selection from the literature search.

Study selection involved title screening, abstract screening, and full‐text screening according to the eligibility criteria. One reviewer (A.A.) independently examined the results returned by the PubMed and CINAHL searches to identify potentially relevant studies. The inclusion criteria involved a diagnosis of breast cancer stages I to III, patients receiving chemotherapy, English text articles, studies published within the last 10 years, and studies that report psychological distress measures. Exclusion criteria included studies in males, non‐breast cancer diagnosis cases, studies including metastatic breast cancer cases, non‐English text articles, qualitative studies, literature review articles, opinion‐based articles, and case reports. Initial screening yielded 529 potentially relevant studies. After duplicates were removed (*n* = 47), a total of 287 were excluded because they were not related to the purpose of this review. The remaining 195 studies were advanced to abstract screening. Among the studies screened, 46 systematic reviews and meta‐analyses were excluded. Furthermore, we excluded 124 searches that mainly lacked the population of interest or the use of chemotherapy. The full‐text screening included 25 studies, and eight articles were excluded. Of these eight studies, five studies were excluded as the sample involved cases of metastatic breast cancer.^29^, ^30^, ^31^, ^32^, ^33^ Three of the remaining studies were excluded because two^34^, ^35^ patients had not been exposed to chemotherapy, and one did not report a psychological distress measure as an outcome.^36^ This review adhered to the Preferred Reporting Items for Systematic Reviews and Meta‐Analyses extension for Scoping Review (PRISMA‐ScR). Thus, there was no risk assessment of bias across the studies in this review.^28^ A flowchart of the inclusion and exclusion of the studies is illustrated in Figure 1.

### 2.4 Charting the data

One author (A.A.) developed draft charting tables to record essential information regarding the operational definition and measures to assess psychological distress. The data were extracted according to the author's name, year of publication, country, study design, sample size, psychological distress measure, operational definition, the timing of distress assessment, stage of breast cancer, and key findings. Since scoping reviews are less likely to evaluate the quality of the studies, we focused on describing the quality of identified instruments to address the scoping review questions.

### 2.5 Collating, summarizing, and reporting of the results

We reported our findings descriptively using tables (See Tables 1 and 2). We analyzed the data by utilizing a narrative synthesis approach. To review the validities and reliabilities of the identified instruments, we adapted the “Guidelines reporting the psychometric soundness of instruments”.^37^ The data were summarized according to the common similarities, themes, and gaps in the literature reviewed. Any modification of the content was discussed and approved by all the researchers.

**TABLE 1:** Description of the included studies.

Author (s)\Year	Country	Sample size	Study design	Stage of breast cancer	Timing of distress measurement	Key findings
Aboalela et al.^40^	United States	*n* = 77	Longitudinal study	Stages I‐III	T1 prior to chemotherapy T2 4 weeks after initial chemotherapy treatment, T3 6 months after initiation of chemotherapy, T4 I year after chemotherapy.	Higher stress levels were noted at baseline (*p* ≤ .0024), stress remained elevated at intermittent times during chemotherapy.
Andreu et al.^43^	Spain	*n* = 102	Longitudinal study	Stages I‐III	Time 1: preliminary diagnosis, Time 2: surgery, Time3: definitive diagnosis, and Time 4: chemotherapy treatment.	38% of women had a clinically significant distress level. Predictors of distress were commonly chemotherapy, helplessness\hopelessness, and anxious preoccupation.
Ando‐Tanabe et al.^38^	Japan	*n* = 18	Longitudinal study	Stages I‐II	Distress measured at two time points (T1 = before chemotherapy), (T2: 4 weeks after final chemotherapy session).	The HADS Anxiety scores were higher in patients with chemotherapy than control at baseline (*p* ≤ .01), and depression scores were high at 4 weeks after chemotherapy (*p* = .071), (*p* = .868), respectively.
Caldeira et al.^48^	Portugal	*n* = 70	Cross‐sectional study	Majority early stages BC	Not applicable.	27% of women have experienced spiritual distress, the main concepts underlying distress were express suffering, crying, anxiety, and alienation.
Cheung et al.^12^	Singapore	*n =* 54	Cross‐sectional study	Stages I to IIIA	Not applicable	Women showed high levels of anxiety, fatigue, and cognitive disturbances.
Henselmans et al.^42^	Netherlands	*n* = 171	Longitudinal study	Stages 0‐III	T1: after diagnosis, T2: after surgery, T3: after radiation and chemotherapy, T4: 2 months from treatment, T5: 6 months after the end of treatment.	Women on chemotherapy showed high distress levels throughout the active treatment period (33%).
Jong et al.^14^	Netherlands	*n* = 83	A randomized control trail	Stages I‐III	Distress measured at T0: baseline, T1: 3 months, T2: 6 months.	Anxiety and depression scores have improved over time in intervention group (yoga) (*p* = .017), (*p* = .031), respectively.
Kardan et al.^13^	United States	*n* = 62	Longitudinal study	Stage 0‐IIIA	Distress measured at three time points (T1: before chemotherapy; T2: 4 weeks after chemotherapy; and T3: 7 months after chemotherapy	Chemotherapy patients reported higher phycological distress at baseline and improved after completion of chemotherapy.
Komatsu et al.^47^	Japan	*n* = 21	A randomized control trail feasibility study	Stage I‐II	Baseline and post intervention.	The self‐directed home yoga program did not significantly improve the psychological distress scores.
Park et al.^15^	Korea	*n* = 117	Longitudinal study	Stages I‐II	Distress assessed at T1: before chemotherapy T2: 1 week post adjuvant chemotherapy and T3: 6 months after adjuvant chemotherapy.	Distress was high at baseline then subsides over time, however, 20% of women had distress throughout the CT. Distress was higher in older women with fear, nervousness, sadness, worry, and loss of interest in usual activities.
Robins et al.^46^	United States	*n* = 145	A randomized control trail	Early‐stage breast cancer	Preintervention: before chemotherapy (Pre‐Ix), within 1 week post intervention (Post‐Ix), 4.5 months (follow up 1: F/u 1), and 6 months after enrollment (follow up2, F/u 2).	Levels of distress were high at baseline (*p* = .0001), patients on the spiritual growth group showed elevated levels of beta‐endorphin immediately post intervention and at 5 months of intervention, no differences were noted in leu‐enkephalin levels among groups.
Sanford et al.^41^	United States	*n* = 80	Longitudinal study	Stages I‐III	Before chemotherapy, mid chemotherapy, and 6 months from initial chemotherapy.	Depressive symptoms were high throughout chemotherapy (*p* = .002), anxiety symptoms were high prechemotherapy and improved overtime (*p* = .04).
Starkweather et al.^39^	United States	*n* = 77	Longitudinal study	Stages I‐III	T1 prior to chemotherapy T2 4 weeks after initial chemotherapy treatment, T3 6 months after initiation of chemotherapy, T4 I year after chemotherapy.	Perceived stress was high at bassline decreased over time (*p* = .001).
Würtzen et al.^45^	Denmark	*n* = 336	A randomized control trail	Stages I‐III	Baseline, immediately after intervention, 6 months from intervention, and 12 months post intervention.	The Mindfulness Based Stress Reduction program improved psychological distress score over chemotherapy at baseline (*p* = .01), at 6 months (*p* ≤ .001), and at 12 months (*p* = .04).
Yang et al.^44^	Taiwan	*n* = 40	Randomized controlled trail	Stages I‐IIIA	Baseline, 6 weeks, and 12 weeks post intervention.	Women in intervention group (home based exercise), showed improvement in distress scores at 6 weeks, and 12 weeks compared to baseline (*p* = .03, *p* ≤ .01), respectively.
Zhang et al.^7^	China	*n* = 88	Longitudinal study	Stages I‐III	T1: first day of the second cycle of Chemotherapy (CT), T2, first day of the third cycle of CT; T3, first day of the fourth cycle of CT, T4, first day of the fifth cycle of CT, T5, first day of the sixth cycle of CT.	Anxiety and depression levels were highest at the first day of the fourth chemotherapy session. Also, symptoms of anxiety and depression persisted throughout the chemotherapy treatment.

**TABLE 2:** Instruments and operationalization of psychological distress.

Reference articles	Operationalization of distress	Distress measures
Aboalela et al.^40^; Starkweather et al.^39^	The degree to which individuals perceive their life stressful over 1 month.	Perceived Stress Scale (PSS): is a ten item Likert Scale Five‐point responses (0 = never, 1 = almost never, 2 = sometimes, 3 = fairly often, 4 = very often).
Andreu et al.^43^	Patients' feelings of somatization, depression, and anxiety.	The Brief Symptom Inventory 18 (BSI‐18) an 18‐item scale rated on five‐point scale reporting patient's distress feeling last 7 days. Reliability: Cronbach's α of 0.79.
Bidstrup et al.^11^; Park et al.^15^	Unpleasant experience of psychological, emotional, spiritual, religious, and social factors that interfere with cancer treatment over a week.	The Distress Thermometer (DT) is a 10‐item scale with 0 no distress and 10 as extreme distress; Problem list (PL), is used to assess predictors of PD. (PL) is a 39 problem list items measuring six domains (practical, family, spirituality \religion, emotional and physical domains). Responses are binary (yes, no).
Caldeira et al.^48^	Spiritual distress as a state of suffering associated with impaired ability of having a meaningful life including (personal, transcendental, communal, and environmental dimensions).	A questionnaire including demographical data, 40 items related to characteristics of spiritual distress according to NANDA‐I. items are rated on binary response (yes, no). The Spiritual Wellbeing Questionnaire (SWBQ). A 20 item Likert Scale including four dimensions (personal, transcendental, communal, and environmental, response rated on five‐point Likert Scale).
Cheung et al.^12^	Subjective symptoms of fatigue, anxiety, depression, and perceived cognitive disturbances. Mental health literacy.	The Beck Depression Inventory (BDI) is a 21‐item tool used to measure the severity of depression within a week. Each item is rated on four‐point scale. A score of 17or more indicates a significant depression. The Beck Anxiety Inventory (BAI) is a 21item instrument measuring anxiety symptoms over 1 month. Each item is rated on four‐point scale. A score of 16or more indicates a significant anxiety. Cronbach's α of 0.92. Test–retest reliability of .75. The Brief Fatigue Inventory (BFI) is a nine‐item instrument measuring the severity of fatigue. Each item is rated on 11‐point Likert Scale from 0 to 10. Fatigue as Moderate (4–6), Sever (7–10) were included in the study. The Functional Assessment of Cancer Therapy‐Cognitive Function (FACT‐Cog) is 37 items measuring cognitive disturbances during last 7 days. Item assessed on five‐point Likert Scale. Cronbach's α of 0.929. The modified mental health literacy Questionnaire is a 47‐item illustrating four cancer specific vignettes each describing the four symptoms of PD. Items were evaluated on five points (0 to 4).
Henselmans et al.^42^	Psychological distress as feeling unable to concentrate, losing sleep, lack of decision making and constant feeling of pressure.	General Health Questionnaire (GHQ‐12). Is a 12‐item scale. Responses rated on four‐point scale (less than usual, no more than usual, rather more than usual, or much more than usual). Reliability: Cronbach's α of 0.84–0.90
Jong et al.^14^; Robins et al.^46^	Subjective distress of dealing with stressful life events.	Impact of Event Scale (IES) 15‐item scale measuring distressing thoughts. Responses are rated on four‐point Likert scale. (0 = not at all, 4 = extremely). Impact of Event Scale (IES‐R) revised version: a 22‐item self‐report measure that assess subjective distress caused by traumatic events.
Kardan et al.^13^	Worry, anxiety, depression, fatigue, and sleep problems.	Worry: three items worry Index (TIWI). Anxiety: Sate Trait Anxiety Inventory (STAI) Depression: Patient health Questionnaire eight (PHQ‐8). Fatigue: Functional Assessment of Cancer Therapy: Fatigue, (FACT‐F). Sleep problems: Pittsburgh Sleep Quality Index (PSQI) Number of items not mentioned.
Sanford et al.^41^; Bidstrup et al.^11^; Komatsu et al.^47^; Jong et al.^14^; Ando‐Tanabe et al.^38^	Emotional distress as a current depressive symptoms and anxiety.	The Hospital Anxiety and Depression Scale (HADS): a 14‐item scale with two sub scales (HADS‐A) anxiety 7 items scale and (HADS‐D) 7 items. Items were rated on four‐point scales (0 = none, to 3 = sever).
Würtzen et al.^45^	Psychological distress symptoms.	The Danish Version of Symptom Checklist 90‐r (SCL‐90r): a 90‐item scale with nine subscales measuring level of distress.
Yang et al.^44^	Emotional distress as mood disturbance examined in six dimensions.	The Profile of Mood States‐Short Form (POMS‐SF), a 30‐item scale measuring a person's psychological distress in six dimensions (tension, anxiety, depression‐dejection, anger‐hostility, vigorous activity). Responses were rated on four‐point Likert scale (0 = not at all, to 4 = extremely). Reliability: Cronbach's α of 0.91–0.94.
Zhang et al.^7^	Depression and anxiety.	Anxiety: Self rating anxiety scale (SAS): a 20 items Likert scale each item rated on four‐point scale. Higher score indicates highest anxiety levels. Cronbach's α of 0.93 Depression: Self rating depression scale (SDS): a 20 items Likert scale each item rated on four‐point scale. Higher score indicates highest depression levels. Self‐report, Cronbach's α of 0.784.

## 3 RESULTS

### 3.1 Primary characteristics of the studies

A total of 17 studies concerning the assessment of psychological distress during chemotherapy were retained for the review. The study designs identified in this review included longitudinal design,^7^, ^11^, ^13^, ^15^, ^38^, ^39^, ^40^, ^41^, ^42^, ^43^ randomized control trials,^14^, ^44^, ^45^, ^46^ a pilot study,^47^ and cross‐sectional studies.^12^, ^48^ The most frequently reported country (*n* = 5) was United States.^13^, ^39^, ^40^, ^41^, ^46^ Two were done in Japan,^38^, ^47^ two in the Netherlands,^14^, ^42^ two in Denmark,^11^, ^45^ one in Korea,^15^ one in China,^7^ one in Singapore,^12^ one in Portugal,^48^ one in Spain,^43^ and one in Taiwan.^44^

The inclusion and exclusion criteria are described in depth in most of the studies (*n* = 14).^7^, ^12^, ^14^, ^15^, ^38^, ^39^, ^40^, ^41^, ^42^, ^43^, ^44^, ^45^, ^47^, ^48^ The sampling types varied among the reports, with random, convenience, and stratified samplings being evident in this review. The data collection sites were commonly single‐site oncology clinics or hospitals, and some data were collected from multiple cancer facilities and population‐based data sources. Also, six studies utilized samples that were secondary data analyses.^11^, ^13^, ^39^, ^41^, ^43^, ^48^ The total subjects of this review were 1864, and the maximum and minimum sample sizes were 18, and 336, respectively.

Most of the studies quantified the attrition rate for completing all the distress assessments throughout chemotherapy and attending the interventions' groups meetings with a retention rate that ranged from 47.3% to 96%. %. Also, the attrition rate among longitudinal studies was 47% to 84%,^11^, ^41^ relatively lower than in randomized trails, at 75% to 86%.^46^, ^47^ For example, Zhang et al.^7^ reported that the total number of the eligible subjects was 102, and only 82 were recruited in the study. Similarly, Sanford et al.^41^ screened 261 potential participants, and only 83 enrolled in the study. The reasons for dropouts were mostly related to family issues, time constraints, transportation issues, and lack of interest in completing the studies' full measurements.

The studies recorded characteristics of the participants that included age, family history of breast cancer, number of children, menopause status, tumor stage, employment status, history of smoking, chemotherapy regimen, and educational attainment. The mean age of women with breast cancer selected in the reviewed studies ranged from 41.67 to 61 years. The population of interest in this review was women with breast cancer who were also undergoing chemotherapy.

The proportion of participants with stage I breast cancer ranged from 10% to 47% while 41% to 73%, had stage II and 3% to 16% had stage III. Most of the studies described the assigned chemotherapy regimens, which commonly included a combination of the following chemotherapy agents: doxorubicin, cyclophosphamide, paclitaxel, epirubicin, fluorouracil, and methotrexate. Two of the longitudinal studies examined the outcomes of distress between the chemotherapy group (*n* = 18; *n* = 20) and a healthy control group (*n* = 22; *n* = 18) or patients receiving other cancer treatments, such as endocrine and radiation therapies.^13^, ^38^ Selection of healthy controls was based on age, negative mammograms, and those who had not undergone any treatment for breast cancer.

The purposes of the studies on psychological distress varied. Most of the longitudinal articles examined the prevalence, patterns, and indicators of psychological distress during chemotherapy.^7^, ^11^, ^15^, ^41^, ^42^, ^43^ Two studies attempted to identify psychological distress symptoms within two different contexts: the mental and spiritual dimensions of the patient.^12^, ^48^

The mental health literacy concept was also utilized to assess patients' abilities to recognize their psychological distress as anxiety, fatigue, depression, or subjective cognitive dysfunction. Another study examined the determinants of spiritual distress among women with breast cancer according to the NANDA‐1 taxonomy II of nursing diagnosis. Furthermore, four studies were related to cognitive disturbances and distress.^12^, ^13^, ^38^, ^39^ Three studies investigated the psychoneurological symptoms cluster and its impact on chromosomal imbalances, systemic inflammation (C‐reactive protein, proinflammatory cytokines), and neuroendocrine‐immune system responses.^39^, ^40^, ^46^ Interventional studies evaluated the efficacy and effectiveness of multiple therapies, including yoga, walking, mindfulness‐based stress reduction (MBSR), tai chi, and spiritual growth groups.^14^, ^44^, ^45^, ^46^ Also among the reviewed reports was a pilot study that evaluated a home yoga program designed to improve cognitive disturbances and distress in patients receiving chemotherapy.^47^

Most of the studies specified the timeframe for the measurement of psychological distress. This varied across the studies from baseline assessment, midpoint of chemotherapy, 6 months from chemotherapy and up to 2 years post chemotherapy. Detailed information on timeframe for measures of psychological distress is described in Table 1.

### 3.2 Instruments for assessing psychological distress

A total of 21 different instruments were utilized to assess psychological distress (See Table 2). Many of the studies used more than one measure to examine psychological distress. Most of the studies used valid and reliable instruments that were pretested in previous literature. Some studies (*n* = 5) reported their internal reliability scores that ranged from 0.75 to 0.94.^7^, ^12^, ^42^, ^43^, ^44^ Most of the reviewed instruments were multidimensional in structure and ranged from one to nine dimensions. The number of items on the tests substantially differed and ranged from 3 to 90 elements. Some of the studies (*n* = 8) reported their cut off points for determining clinically significant cases of psychological distress.^7^, ^11^, ^12^, ^15^, ^41^, ^42^, ^43^, ^47^ The remaining studies discussed how higher scores are indicative of more severe distress levels.

Five studies used the Hospital Anxiety and Depression Scale (HADS), which is a reliable and valid generic questionnaire developed for detecting symptoms of anxiety and depression in patients receiving care at hospitals over 1 week. Although HADS is designed primarily for assessing anxiety and depression, it was used among these studies to describe psychological and emotional distress.^14^, ^38^, ^41^, ^44^, ^47^ Two studies measured psychological distress using the Impact of Event Scale (IES), which is a 15‐item scale that measures subjective distress symptoms that result from cancer experience.^14^, ^46^ Two articles used the Distress Thermometer (DT), a 10‐item visual analogue scale in which a score of 0 indicates no distress, while 10 indicates extreme distress. The DT also incorporates a Problem List (PL), which is a set of 39 problems that may produce distress. The PL includes five domains (physical, practical, familial, emotional, and spiritual and religious). Two studies measured stress levels with the Perceived Stress Scale (PSS), which is a ten‐item Likert scale that measures the perception of stress over 1 month.^39^, ^40^

The remaining studies each used different methods for measuring psychological distress. They included the following scales: the Brief Symptom Inventory 18 (BSI‐18), the 12‐Item Version of General Health Questionnaire (GHQ‐12), the Three Items Worry Index (TIWI), the State–Trait Anxiety Inventory (STAI), Patient Health Questionnaire‐8 (PHQ‐8), the Functional Assessment of Cancer Therapy: Fatigue, (FACT‐F), the Pittsburgh Sleep Quality Index (PSQI), Symptom Checklist 90‐r (SCL‐90r), The Profile of Mood States‐Short Form (POMS‐SF), the Beck Depression Inventory (BDI), the Beck Anxiety Inventory (BAI), the Brief Fatigue Inventory (BFI), the Functional Assessment of Cancer Therapy‐Cognitive Function (FACT‐Cog), The Modified Mental Health Literacy Questionnaire, the Spiritual Wellbeing Questionnaire (SWBQ), and a Questionnaire of Nursing Diagnosis of Spiritual Distress.

The methods of data collection included self‐report (*n* = 18), interviews (*n* = 3) DT, FACT‐F, and questionnaires to determine a NANDA diagnosis of spiritual distress and mental health. Mostly Likert scales (*n* = 21) were used; one was a visual analogue scale (DT), and one was a binary scale (PL). The majority of measures were generic (*n* = 16), but four were explicitly developed for cancer (DT, BSI, FACT‐F, and FACT‐Cog). FACT‐F is a measure of fatigue for cancer patients; the researchers used it as an indicator of psychological distress along with other measures, such as PHQ‐9 and STAI.^13^ Also, Cheung et al.^12^ used the FACT‐Cog to measure the subjective cognitive disturbances among BCSs as a type of psychological distress. Two questionnaires were designed to examine the characteristics of distress (psychological and spiritual). Further details of the reviewed instruments are presented in Table 2.

### 3.3 The concepts defining psychological distress

**FIGURE 2:**
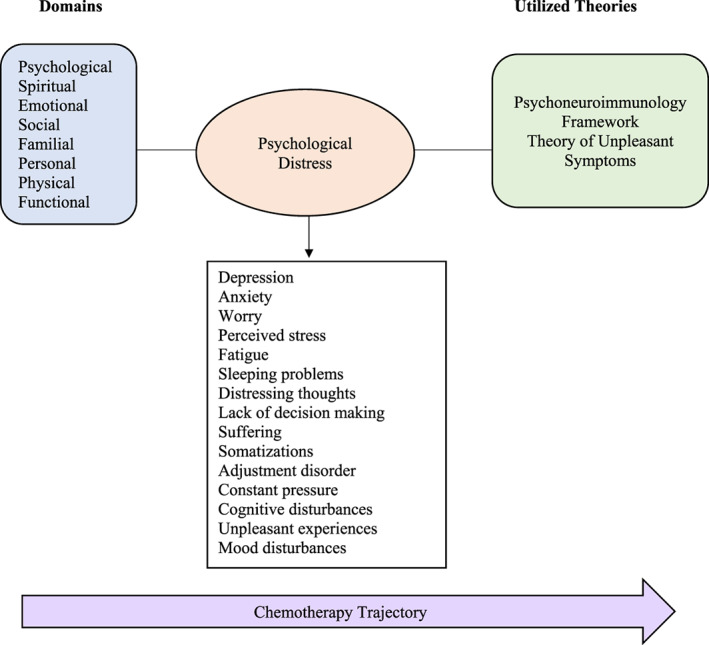
A Conceptual map of related concepts and constructs of psychological distress in reviewed studies.

More than 15 concepts and constructs were utilized to define psychological distress. Studies that aimed to identify distress or its predictors mainly examined concepts of depression and anxiety. However, some studies examined psychological distress in a broader context. These studies intended to explore the associated factors, predictors, and symptoms of psychological distress and used terms that included: “distress (spiritual, emotional),” “worry,” “anxiety,” “perceived stress,” “fatigue,” “sleeping problems,” “distressing thoughts,” “lack of decision making,” “suffering,” “somatizations,” “adjustment disorder,” “constant pressure,” “cognitive disturbances,” and “unpleasant experiences that interfere with cancer treatment.” The range of the reviewed domains of the instruments was between 2 and 14. The psychological and emotional domains were the most prominent. Other areas were related to social, spiritual, familial, personal, physical, biological, and functional categories. The reviewed studies' identified concepts and constructs are illustrated in Figure 2.

Among the reviewed studies, there were a few (*n =* 6) that utilized theoretical frameworks to guide the development of research on psychological distress. The reported frameworks were the Theory of Unpleasant Symptoms (TUS) (*n* = 2)^7^, ^44^ and (*n* = 4) the Psychoneuroimmunology Framework (PNI).^39^, ^40^, ^41^, ^46^ The TUS is based on the premise that the physical, conditional, and psychological factors are antecedents that impact the experience of symptoms.^44^ Psychological concepts of distress within this framework included anxiety, depression, and mood disturbances. The PNI framework is based on the underlying pathways of psycho‐behavioral neuroeducation‐immune system processes.^39^, ^46^ Psychological distress was conceptually differentiated within the PNI framework as perceived stress, depression, and anxiety.

## 4 DISCUSSION

A scoping review was undertaken to examine the literature for the assessment of psychological distress among BCSs. Generally, the studies measured psychological distress during chemotherapy and evaluated the effectiveness of interventions meant to alleviate symptoms of psychological distress. Within this scoping review, we identified several studies that utilized various measures for psychological distress, mainly the HADS, DT, PSS, and IES. Most of these instruments were self‐rated scales. Thus, the information obtained is subject to recall bias, which affects the generalizability of the study findings.

Moreover, among the reviewed studies, there was considerable variation in the use of generic or cancer‐specific instruments. The generic tools such as HADS, BDI, PHQ‐8, GHQ‐12, and STAI were utilized more than the cancer‐specific measures. Although HADS is a well‐validated tool that was initially developed to assess anxiety and depression levels among physically ill patients,^47^ it may not precisely measure psychological distress. HADS had two subscales, each measuring a different concept. Seven items measure symptoms of depression, and seven items are related to anxiety. The overall score represents the total levels of anxiety and depression.^47^ Since HADS is limited to measuring the symptoms of depression and anxiety, it may misinterpret the underlying causes of psychological distress. Similarly, the NBCC report of psychological distress illustrates that the HADS is only applicable for screening anxiety, not distress.^25^ Thus, HADS may not be the best instrument to detect psychological distress among women with breast cancer.

The cancer‐specific scales included two that were not designed to measure distress (FACT‐F and FACT‐Cog). The Functional Assessment of Cancer Therapy: Fatigue (FACT‐F) is designed to assess fatigue among cancer patients,^13^ and the Distress Thermometer was developed explicitly to detect distress among cancer patients. The DT is a short visual analogue scale. Patients who reported high levels of distress were asked to relate possible causes of distress, according to the Problem List (PL). But the lack of agreement on utilizing a specific distress measure for cancer patients may lead to inconsistent findings of psychological distress.

The reviewed studies differed in explaining the scores needed to distinguish between psychological distress present cases and no distress cases. There were studies that emphasized reporting the cutoff points of psychological distress based on the purpose of the instrument.^39^, ^42^, ^43^ We also found studies that did not describe their method to determine high distress levels or cases of psychological distress.^38^, ^45^ For example, the NCCN recommends using a score of 4 as a specific indicator for distress cases, but we found different cutoff points reported (4, 7) for using the DT.^11^, ^15^ Thus, the diversity of interpretation methods and the significant variations in cutoff scores for determining cases of psychological distress might hinder the detection of psychological distress within the population.

In this review, reports on the reliability or validity of the instruments were scant. The reliability or validity methods were not explicitly stated. Few studies specified the procedures for examining the reliability, which included Cronbach alpha and test‐rest reliability. For example, Zhang et al.^7^ described the internal consistency of both the Self‐rating Anxiety Scale (SAS) and Self‐rating Depression Scale (SDS) as 0.93 and 0.78, respectively. However, the study did not provide the validity of both scales. Similarly, Andreu et al.^43^ indicated that the internal consistency of the BSI‐18 was 0.79. Test–retest reliability was used to evaluate the BAI, resulting in a value of 0.75.^12^

Reporting on the validity of the instruments was also lacking. According to Devon et al.^37^ a tool is valid if it accurately reflects meaning of a particular phenomenon under study. There are several types of validation, including construct validation, face validation, and content validation.^37^ Some of the reviewed studies explained earlier validation strategies (i.e., construct and content validity) within breast cancer patients.^11^, ^43^, ^44^, ^46^

Collectively, the reviewed studies lacked detailed descriptions of the reliability, and validity of the instruments utilized, which are necessary elements in gathering reliable and accurate data. Also, it is essential to validate the generic tools within the population of breast cancer to specifically examine psychological distress.^37^ Therefore, the applicability of the generic scales needs a further validation process among breast cancer patients.

Although most of the studies defined psychological distress as depression and anxiety, usage of the term varied among the studies. We found several words that were used to describe distress, including: “worry,” “stress,” “fatigue,” “cognitive disturbances,” “sleep disturbances,” and “mood disturbances.” Furthermore, within the conceptualizations, we found studies that described psychological distress through five categories. For instance, Kardan et al.^13^ conceptualized psychological distress as worry, anxiety, depression, fatigue, and sleep disturbance. Another study by Cheung et al.^12^ examined cognitive disturbance and other symptoms as distress. These variations of terms describing psychological distress could be related to symptoms that commonly co‐occur with distress in women with breast cancer. Moreover, we noted a heterogeneity in empirical indicators of the concept of perceived stress as it was tested by two different scales (IES and PSS).^39^, ^40^, ^46^ Hence, the lack of clarification in defining psychological distress results in its being misrepresented in the literature.

Psychological distress was structured mostly as a multi‐dimensional construct. The studies reported multiple domains, mainly mental and emotional ones. Other categories related to individual, social, spiritual, and functional factors. For instance, Kardan et al.^13^ examined the domain of sleeping and fatigue in assessing psychological distress, while five studies examined solely the mental and physical domain.^14^, ^38^, ^40^, ^41^ The variations in assessing the domains of psychological distress may lead to confusing outcomes. Thus, future studies should examine the collective concepts and areas underlying the construct of psychological distress. Future research should also focus on unifying the specific dimensions of distress to enable scholars to accurately assess psychological distress.

The conceptualization of psychological distress was not adequately supported by a theoretical framework. Few studies utilized theoretical models to examine psychological distress. Both Robins et al.^46^ and Starkweather et al.^39^ explored distress using the Psychoneuroimmunology Framework (PNI), while Zhang et al.^7^ and Yang et al.^44^ used the Theory of Unpleasant Symptoms. In their study, Yang et al. developed an intervention to mitigate psychological distress. The study also operationalized psychological distress within six dimensions (tension, anxiety, depression‐dejection, anger‐hostility, and vigorous activity), but these dimensions only represent the psychiatric and emotional domain. Overall, the varying constructs and domains of conceptualizing psychological distress still lack a unified theory.

### 4.1 Study limitations

This review focused specifically on survivors of early‐stage breast cancer who are undergoing chemotherapy. We emphasize the need for comprehensive assessment, including different populations of all cancer types. Furthermore, our review is limited to English text studies, and countries tend to develop measures in their native language. Our search only covered two databases and excluded literature reviews. This was done to overcome the challenges that might exist in managing the data related to the broadness of distress within literature reviews and only limit this review to include original articles that report measures of psychological distress. Furthermore, in keeping with PRISMA‐ScR definition of scoping reviews, critical appraisal of quality for each included study was not part of this scoping review's methodology.^28^ In this review, only a narrative synthesis method was used in reporting the findings. Therefore, the results are subject to reporting bias, as it possible that the results may lack several studies that could be relevant to the review's aim.

## 5 CONCLUSION

This review highlights the gaps in the operationalization and instruments used to measure psychological distress among breast cancer patients during chemotherapy. We found 21 measures for assessing psychological distress and were self‐reported scales. The common instruments reporting psychological distress in the reviewed studies were mainly HADS, DT, PSS, and IES. The inclusion of reliability and validity indicators for measuring distress was insufficient in the reviewed studies. Future research should incorporate detailed information on psychometric properties to increase the trustworthiness of findings.

The findings described the various conceptualizations of the term psychological distress (anxiety, stress, worry, mood disturbances, depression, fatigue, sleep problems, and cognitive distress). Each concept and measure indicate the complexity of unifying the term psychological distress within the chemotherapy treatment trajectory. This is likely to result as patients during chemotherapy treatments deal with multiple distressing psychological symptoms such as depression, sleep disturbances, and fatigue. However, the identified concepts are unique to breast cancer patients' distress experience during chemotherapy, and this elaborate and provides insights on how these concepts enhance nurses' understanding of psychological distress.

This review concluded that the lack of a standard definition and comprehensive measure of psychological distress might limit the identification of distress in women with early‐stage breast cancer. This supports previous evidence on the underassessment of psychological distress.^19^, ^20^ Therefore, additional research is needed to examine the collective meaning of the identified concepts for predicting and measuring psychological distress in a broader set of patients from other cancer populations.

This review provides valuable information for nurses, researchers and clinicians caring for individuals with breast cancer. As per current practice recommendations, early identification of psychological distress is essential in enhancing distress management in women diagnosed with breast cancer.^18^ Therefore, oncology nurses should use validated cancer specific measures such as the distress thermometer. The identified instruments and concepts may guide researchers toward developing or modifying existing instruments to manage psychological distress among breast cancer survivors.

## AUTHOR CONTRIBUTIONS

**Amal Khualif Alanazi:** Conceptualization (lead); methodology (lead); writing – original draft (lead). **Debra Lynch‐Kelly:** Writing – review and editing (supporting). **Michael Weaver:** Writing – review and editing (supporting). **Debra E. Lyon:** Conceptualization (supporting); writing – review and editing (supporting).

## CONFLICT OF INTEREST STATEMENT

The authors have stated explicitly that there are no conflicts of interest in connection with this article.

## ETHICS STATEMENT

Not applicable.

## Supporting information


**Data S1:** Supporting Information

## Data Availability

Not applicable.
