# Supplementary material for: A scoping review of psychological distress instruments in women with early‐stage breast cancer during chemotherapy
Source: Cancer Rep (Hoboken). 2023 May 12;6(6):e1833. doi: 10.1002/cnr2.1833 (PMC10242653; doi:10.1002/cnr2.1833)
Supplement: Supplementary file 1 — Data S1: Supporting Information [file CNR2-6-e1833-s001.docx]

**Supplemental File 1**

**Scoping Review Search Strategy**

PubMed Search Strategy

The PubMed database was searched in May 2019 for the articles published over the last ten years. The search strategy was as follows: ("Stress, Psychological"[Mesh] stress [Text Word] OR distress [Text Word] AND ("Drug Therapy"[Mesh] OR "drug therapy"[Subheading] OR chemotherapy [Text Word] OR “drug therapy” [Text Word] OR “active treatment” [Text Word]) AND ("Breast Neoplasms"[Mesh] OR "breast cancer"[Text Word] OR "breast neoplasm"[Text Word] OR "breast tumor"[Text Word] OR "breast carcinoma"[Text Word]).

CINAHL Search Strategy

The CINAHL database was searched in May 2019 for the articles published over the last ten years. The search strategy was as follows: "distress" OR "stress" OR (MH "Stress, Psychological”) AND (MH "Chemotherapy, Cancer") OR (MH "Chemotherapy, Adjuvant") OR (MH "Antineoplastic Agents, Combined") OR (MH "Neoadjuvant Therapy”) AND (MH "Breast Neoplasms") OR "breast cancer".
